# An Efficient Strategy Developed for Next-Generation Sequencing of Endosymbiont Genomes Performed Using Crude DNA Isolated from Host Tissues: A Case Study of *Blattabacterium cuenoti* Inhabiting the Fat Bodies of Cockroaches

**DOI:** 10.1264/jsme2.ME14153

**Published:** 2015-07-04

**Authors:** Yukihiro Kinjo, Seikoh Saitoh, Gaku Tokuda

**Affiliations:** 1 Tropical Biosphere Research Center, University of the Ryukyus, Senbaru 1, Nishihara, Okinawa 903–0213, Japan; 2 Graduate School of Engineering and Science, University of the Ryukyus, Senbaru 1, Nishihara, Okinawa 903–0213, Japan; 3 Japan Collection of Microorganisms/Microbe Division, RIKEN BioResource Center, Tsukuba, Ibaraki 305–0074, Japan; 4 Graduate School of Bioscience and Biotechnology, Tokyo Institute of Technology, Meguro, Tokyo 152–8550, Japan

**Keywords:** Endosymbiont, genome assembly, gap closing, targeted genome reconstruction, bacterial gene identification

## Abstract

Whole-genome sequencing has emerged as one of the most effective means to elucidate the biological roles and molecular features of obligate intracellular symbionts (endosymbionts). However, the *de novo* assembly of an endosymbiont genome remains a challenge when host and/or mitochondrial DNA sequences are present in a dataset and hinder the assembly of the genome. By focusing on the traits of genome evolution in endosymbionts, we herein developed and investigated a genome-assembly strategy that consisted of two consecutive procedures: the selection of endosymbiont contigs from an output obtained from a *de novo* assembly performed using a TBLASTX search against a reference genome, named TBLASTX Contig Selection and Filtering (TCSF), and the iterative reassembling of the genome from reads mapped on the selected contigs, named Iterative Mapping and ReAssembling (IMRA), to merge the contigs. In order to validate this approach, we sequenced two strains of the cockroach endosymbiont *Blattabacterium cuenoti* and applied this strategy to the datasets. TCSF was determined to be highly accurate and sensitive in contig selection even when the genome of a distantly related free-living bacterium was used as a reference genome. Furthermore, the use of IMRA markedly improved sequence assemblies: the genomic sequence of an endosymbiont was almost completed from a dataset containing only 3% of the sequences of the endosymbiont’s genome. The efficiency of our strategy may facilitate further studies on endosymbionts.

Numerous insects have established intimate mutualistic relationships with microorganisms, which play key roles in helping the hosts adapt to various environments (7). Specifically, 10%–20% of all insects are estimated to harbor endosymbiotic bacteria within specialized cells called bacteriocytes (7). These intimate associations between insects and endosymbionts have co-evolved as obligate symbioses over several hundred million years (27). These bacterial endosymbionts cannot be cultivated when isolated from bacteriocytes because the bacteria have lost the genes that are essential in free-living forms. Therefore, the genomic sequences of these bacteria have been analyzed, and the findings obtained have contributed substantially to the elucidation of their biological functions in the evolutionary histories of the host organisms (11, 28, 37, 42). A powerful tool currently available for the purpose of genomic analysis is whole-genome sequencing performed using next-generation sequencing (NGS) technology.

Prior to extracting biological information from sequence data, large numbers of short reads obtained from an NGS run must be assembled *in silico* into contiguous sequences (the so-called contigs) of the genome of the organism of interest (*de novo* assembly). However, a problem that is frequently encountered in this computational step is that multiple sequences (*i.e.*, contigs) are obtained that are not equivalent, in terms of number and length, to the bacterial genome, but are myriad shorter contigs. These contigs, particularly in the case of an endosymbiont, typically include not only sequences from the bacterial genome, but also those from other genomes such as host and mitochondrial genomes. Consequently, a large amount of time must be spent identifying the origin of each of these numerous contigs and then performing additional gap-closing procedures by Sanger sequencing (40).

The aforementioned problem occurs when a sequence-assembly program cannot completely assemble a genome sequence. Most assemblers employ mathematical graphs of various types to construct a genome sequence from sequence reads. However, even when DNA obtained from an isolated or purified genome is sequenced, if it contains multi-copied genes and/or repetitive elements in chromosomes, the mathematical graph will be complex (because of the so-called ‘branching’ of paths; reviewed in 25, 31), and, thus, separate contigs will be present in the output (15, 25, 31, 41). When sequencing an endosymbiont’s genome, the presence of reads derived from host and mitochondrial genomes makes the results of *de novo* assembly markedly more complex because the sequences obtained from these other genomes, which are partially similar to the sequences of the target genome, will increase the complexity of the mathematical graph.

Since the presence of extraneous reads derived from other genomes in a dataset is a major cause for the unsuccessful assembly of an endosymbiont genome, the following two steps may improve the genome-assembly results: (1) Selecting (and thus separating) the contigs derived from the target endosymbiont genome from the contigs derived from mitochondrial or host genomes after performing *de novo* assembly. (2) Filtering the entire dataset of reads based on the selected contigs to remove reads from other genomes and reassembling the contigs using the cleaned dataset.

To implement our sequence-assembly strategy, we developed a homology search based on the amino acid sequences of the coding genes of related genomes as a method of contig selection for an endosymbiont. By assuming that the set of genes harbored in an obligate endosymbiont’s genome, which has shrunk in size due to gene loss during its history of symbiosis, was a subset of all genes in their free-living common ancestor and that the amino acid sequences of these genes were highly conserved (32, 38, 44), we hypothesized that even the genomic sequence of a distant free-living relative of an endosymbiont-of-interest may be used as a reference genome in the search for endosymbiont contigs. Furthermore, when this method of contig selection is implemented based on the sequences obtained, it will be possible to filter out reads derived from other genomes that adversely affect sequence assembly. Moreover, reassembling the contigs obtained from the cleaned reads will improve the sequence assembly over its previous assembly.

In order to examine the hypotheses underlying our strategy, we herein evaluated several methods of contig selection in terms of their accuracies and sensitivities in detecting sequences derived from a target genome. As a comparison, we used two reference-based methods (employing nucleotide or amino acid sequence similarities as a reference) with four distinct reference genomes genetically distant from the target endosymbiont to differing extents (from the strain to phylum level; *i.e.*, eight combinations in total between methods and reference genomes), and a reference-independent method (based on sequence composition and the depth of coverage) suggested previously (16). Furthermore, we determine whether and how reassembling contigs obtained from filtered reads using the selections improved assembly with increasing iterations, with respect to both the number of contigs and coverage of the genome sequence by the contigs. Moreover, the effects of the size of the input dataset were evaluated in the aforementioned procedures. In order to implement this strategy, we sequenced two genomes of *Blattabacterium cuenoti*, an obligate bacterial intracellular symbiont that is harbored by almost all cockroaches and the most primitive termite (3, 4, 6, 10, 20, 21), isolated from two cockroach subspecies, *Panesthia angustipennis spadica* and *Panesthia angustipennis yayeyamensis*. This endosymbiont exists exclusively in the bacteriocytes of the fat bodies of the cockroach, in which no other symbiotic bacteria are co-localized (6, 21). Based on the results obtained, we discussed the significance of our strategy.

## Materials and Methods

### Sample preparation

Cockroaches belonging to the two subspecies *P. angustipennis spadica* and *P. angustipennis yayeyamensis* were collected on Mt. Tsukuba (E140°06′24, N36°13′31), located on Honshu Island on the Japanese mainland, in January 2011, and on Iriomote Island (E123°51′43, N24°17′33) in the Ryukyu Islands in September 2011, respectively. Cockroaches were identified to the subspecies level based on the morphological traits of their exoskeletons according to Asahina (1), and these identifications were also confirmed via homology searches of the mitochondrial 12S ribosomal RNA (rRNA) gene sequences against the NCBI nucleotide collection (NT) database. Collected cockroaches were reared until use at room temperature with the logs they had inhabited at the sampling locations. Hereafter, *B. cuenoti* strains obtained from *P. angustipennis spadica* and *P. angustipennis yayeyamensis* were referred to as strains BPAA and BPAY, respectively. Total DNA was extracted from the fat body dissected from a single female cockroach for each endosymbiont strain. DNA was extracted using Isoplant II (Nippon Gene, Tokyo, Japan) and purified using DNeasy Plant Mini Kit columns (Qiagen, Hilden, Germany). The shotgun NGS libraries of the strains (hereafter referred to as BPAA and BPAY libraries) were prepared using TruSeq DNA Sample Prep Kits (Illumina, San Diego, CA, USA) together with distinct multiplex identifier (MID) tags. The insert sizes in the two libraries, which were estimated in a subsequent *in silico* analysis, were approximately 230 nucleotides (nt). In all of these procedures, we followed the manufacturer’s instructions.

### Sequencing and quality filtering of raw data

The libraries were sequenced concurrently using one lane of an Illumina HiSeq2000 sequencer. The sequencers’ raw-read outputs were filtered using the FASTX-Toolkit (http://hannonlab.cshl.edu/fastx_toolkit) as follows: Bases that had Phred quality scores <27 were trimmed from the 3′ end and, after trimming, the reads that had an overall mean <27 and length <80 nt were discarded. No mismatch was allowed in adapter sequences. Unpaired single reads were excluded using CMPFASTQ_PE (http://compbio.brc.iop.kcl.ac.uk/software/cmpfastq_pe.php). Hereafter, the quality-filtered reads were referred to as ‘reads’.

### *De novo* assembly tools

In order to determine the sequence assembler software to be used in the present strategy (to be explained later) to assemble genomes from the present sequence datasets, we examined three *de novo* assemblers: Newbler (Roche), ABySS (43), and IDBA-UD software (34), which implement distinct algorithms. The settings used for the assemblers were as follows. In Newbler, an assembler based on the overlap layout consensus (OLC) algorithm, a ‘-large’ option was specified (equivalent to the ‘large or complex genome’ option in the GUI mode), which reduced the computational time when numerous reads derived from the host genome were present in a dataset. In the ABySS assembler, a De Bruijn graph-based assembler, we set the length of k-mer to 60 (*i.e.*, approximately two thirds of the sequence length of a read in the present datasets). In IDBA-UD, another assembler based on De Bruijn graphs, the default parameters were used. This assembler constructs multiple mathematical graphs with different k-mer sizes for a genome assembly.

The quality of the output from each assembler was evaluated by QUAST (12), a quality assessment tool for genomic sequence assemblies. This assessment was based on two assembly statistics outputs from QUAST, ‘NGA50’ and the ‘genome fraction’. NGA50 was defined as the length of the contig that corresponded to the weighted mean of the length of the reference genome size (in this study, we used the complete genomes of BPAA and BPAY, whereas estimated values are often used in other studies), and was calculated from the contigs that aligned onto the complete reference genome. The genome fraction was the portion of the reference genome aligned by contigs.

### Strategy for assembling the genomic sequence of endosymbionts

**Fig. 1:**
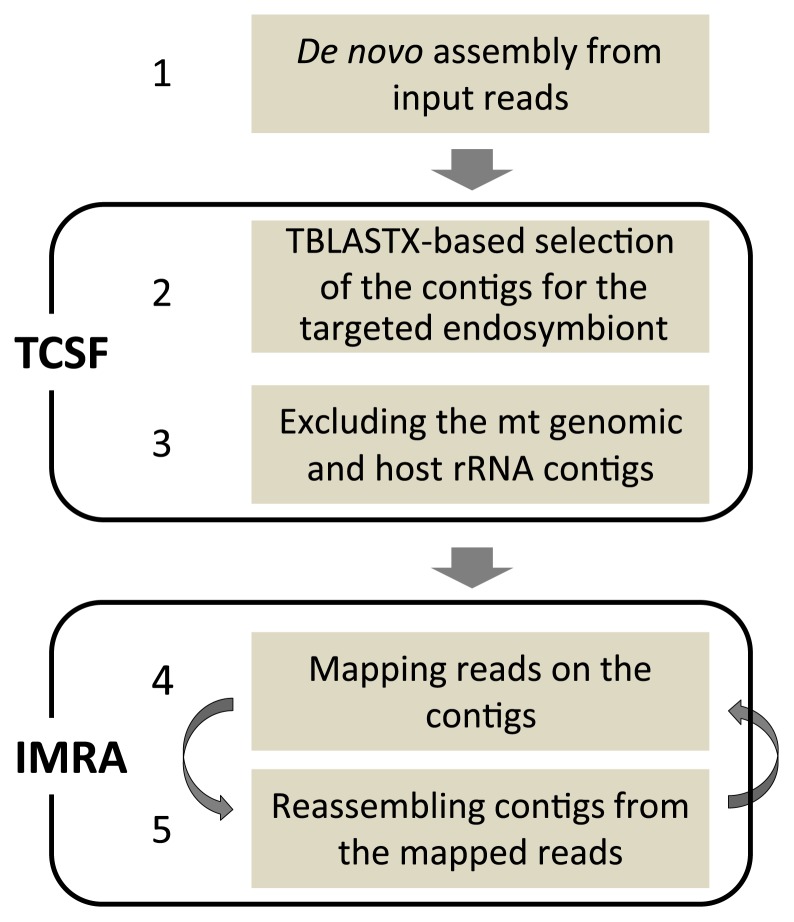
Flowchart of the sequence-assembly procedure developed in this study. (1) *De novo* sequence assembling from input reads, performed in the paired-end mode by using Roche Newbler software. (2) Selecting contigs for endosymbionts by performing TBLASTX searches (E<10^−12^) against the genomic sequence of a related genome. (3) Filtering the selected contigs by using BLAST to exclude contigs obtained from mitochondrial genomes (*i.e.* contigs with higher scores to mitochondrial genomes than to the related genome by using TBLASTX, E<10^−12^) and rRNA genes derived from the host genome (by using BLASTN, E<10^−12^). (4) Mapping input reads onto the filtered contigs by using the Roche Reference Mapper software in the single-end mode. (5) Reassembling contigs from the mapped reads by using the paired-end mode (minimal length of contigs: 500 nt). Iterations of (4) and (5) are continued until the increase in the total length of contigs and the reduction in the number of contigs reach saturation.

The strategy developed in this study for assembling the genomic sequences of endosymbionts comprised 5 steps (Fig. 1):

In the first step, all (or a part) of the reads were assembled *de novo* using the Newbler assembler (Ver. 2.9) in a paired-end mode with default settings. In this study, we excluded contigs shorter than 100 nt.

TBLASTX Contig Selection and Filtering (TCSF; 2^nd^ and 3^rd^ steps in Fig. 1): In the first step in TCSF (2^nd^ step in Fig. 1), the output contigs were examined by performing local TBLASTX searches (NCBI LOCAL BLAST+ software; ref. 8, using default settings, with the exception of num-alignment=1 and a cut-off threshold of E<10^−12^) against the genomic nucleotide sequence of a related organism, after which contigs without any hits were filtered out. In the next step (3^rd^ step in Fig. 1), the remaining contigs were filtered further by performing BLAST searches against two types of sequences, the mitochondrial genome sequence (NC_012901; by using TBLASTX, E<10^−12^) and rRNA gene sequence (AF005243; by using BLASTN [using the megablast algorithm; hereafter referred to as BLASTN], E<10^−12^) from *Blattella germanica*, which is a cockroach belonging to a different family from *P. angustipennis*; the contigs that retrieved better hits to these sequences than to the targeted genome were excluded.

Iterative Mapping and ReAssembling (IMRA; 4^th^ and 5^th^ steps in Fig. 1): The reassembling procedure comprised two steps. The first step (4^th^ step in Fig. 1) was the mapping of all input reads onto the selected contigs using the non-paired-end mode of Roche Reference Mapper software (Ver. 2.9) with default parameters. The second step (5^th^ step in Fig. 1) was the reassembling of the contigs using the paired-end mode of the software from the mapped reads (which were registered as either ‘Full’ or ‘Partial’ by the software). In this step, any read-pairs including at least one read that was mapped (fully or partially) onto the selected contigs were collected from the dataset; they were then input into the new assembly, thereby contributing to the elongation and/or merging of contigs. The first and the second steps (4^th^ and 5^th^ steps in Fig. 1) were then iteratively repeated until the total (increasing) length and total (decreasing) number of contigs had both been saturated. In IMRA, contigs shorter than 500 nt were discarded.

### Assessment of TCSF and IMRA performance quality

In order to validate the TCSF procedure with a focus on the effects of using TBLASTX, we compared TCSF with a method in which the BLASTN program was used to select contigs from a reference genome (E<10^−12^). Furthermore, to evaluate the effects of the genetic distance between the target and reference genomes used in TCSF or BLASTN, we used four genomic sequences that varied in genetic distance from the target endosymbiont (Table 1): the most closely related *B. cuenoti* strain BGIGA (belonging to the same species as the target genomes, harbored by *Blaberus giganteus*, NC_017924, ref. 13; for sequence similarity to the target genome, see Table S1), a closely related free-living *Flavobacterium johnsoniae* strain UW101 (belonging to a different family in the same order, ATCC 17061, NC_009441, ref. 24) and two distantly related strains, *Bacteroides fragilis* strain 638R (belonging to a different class in the same phylum, NC_016776, ref. 33) and *Bacillus subtilis natto* strain BEST195 (belonging to a different bacterial phylum, NC_017196, ref. 30). Refer to Table 1 for detailed information on the genomes and taxonomy of these organisms. Genetic distances among the *B. cuenoti* strains were evaluated by aligning the whole genome using NUCmer (19). Moreover, as a comparison, we employed Concoct (16), a recently developed reference-independent method, which clusters contigs assembled from metagenomics datasets into species-levels by utilizing their sequence statistics (composition of k-mers and depth of coverage). The selection of contigs by Concoct for the present datasets was performed with default parameters, with the exception that the “number of clusters” was set to three since the datasets were expected to contain reads derived mainly from three genomes (endosymbiont, host, and mitochondrion).

We examined these methods of contig selection in terms of sensitivity (*i.e.*, how many contigs derived from the target genome were included and excluded by the selection: True Positive [TP] and False Negative [FN], respectively), and accuracy (*i.e.*, how many contigs derived from other genomes were incorrectly included; False Positives, FP); these were evaluated in terms of the total length of the contigs. In the aforementioned procedures, a contig was regarded as being derived from the target genome or from other genomes based on the criterion of whether its sequence was >99.0% similar to the completed genome sequence (NCBI NC_020510 and DDBJ AP014609 for *B. cuenoti* strains BPAA and BPAY, respectively). This threshold of similarity was determined based on the distribution of percent identities in the results of BLASTN searches.

In order to evaluate how IMRA improved sequence assembly with increasing iteration, the IMRA procedure was iterated beginning with a filtered set of contigs obtained from a *de novo* assembly performed using TCSF, and the total length and number of contigs were recorded as statistics after each cycle of iteration. This procedure was performed using distinct numbers of input reads and different reference genomes (Table 1) to evaluate the effects of data size and genetic distance between target and reference genomes.

Furthermore, we compared the performance of IMRA on the present datasets with that of PRICE (36), a tool based on a similar idea to IMRA (albeit differently implemented), to examine its utility. In order to achieve a fair comparison, two parameters common to both methods, ‘minimum overlap identity’ (-mi flag in Newbler, the assembler and mapper used in IMRA, and -mpi flag in PRICE) and ‘minimum overlap length’ (-ml flag in Newbler, -mol flag in PRICE) were set to the same values. We examined two settings for each of these parameters, in the form of parameter set A (90 and 40 for minimum overlap identity and minimum overlap length, respectively) and set B (85 and 35). The former set represents the default values in Newbler and the latter those in PRICE.

In our testing procedures, the numbers of reads input for the BPAA and BPAY libraries were 0.15–0.5 and 1.5–5 M, respectively; these numbers were selected so that the input datasets contained depths of coverage ranging from approximately 10× to 50× in the case of the target genomes. The portion of the reads derived from the target endosymbiont in a dataset was estimated by mapping reads onto the complete genomic sequence of each strain (hereafter, this portion was referred to as “purity”).

In order to validate the entire strategy used in this study, which consisted of *de novo* assembly, TCSF, and IMRA, the approach was applied on the BPAA and BPAY libraries by using a free-living distant relative (*Bacteroides fragilis*) as a reference genome. We determined whether the quality score per base (Phred quality score) exceeded 60 for 99.99% of the bases of the entire set of contigs to validate the final output contigs obtained from each of the trials. In the process of annotating the newly completed genomic sequence of *B. cuenoti* strain BPAY, the sequence was chimera-checked by performing BLAST searches against public databases and by using dot-plots to compare the sequences to all related genomes that have been sequenced.

All the procedures described herein were performed using in-house Perl, R, and Shell scripts. Parameters for the software tools used in the present strategy and comparative analyses are given in Supplemental file 1. The source codes of TCSF and IMRA, documentation, and example sequence data are available at http://www.cc.u-ryukyu.ac.jp/~tokuda/seqdata_and_script/.

The *B. cuenoti* strain BPAA dataset described here was used in our previous study in which the complete sequence of the genome was reported (ref. 46; NCBI NC_020510), whereas the *B. cuenoti* strain BPAY dataset was newly sequenced in this study. The sequence data obtained in this study have been submitted to the DDBJ Sequence Read Archive (DRA) under accession nos. DRA002289 and DRA002308. The sequence of the complete genome of *B. cuenoti* strain BPAY has been submitted to the DDBJ under accession no. AP014609.

## Results

In Fig. 1, we schematically depicted the strategy tested in this study, which comprised the steps of *de novo* assembly, TCSF, and IMRA. Newbler was used for all assembling procedures because it was selected as the most appropriate *de novo* assembler for the present sequence datasets from the three tested, as described below. In order to evaluate the performance of TCSF as a method of selecting the contigs of target genomes from the output of *de novo* assembly, we compared the sensitivities and accuracies of methods that were nucleotide based (BLASTN), translated-sequence based (TCSF), and reference independent (Concoct). We then evaluated the performance of IMRA in improving assembly with increasing iterations performed using distinct initial contig sets obtained from the preceding step (*i.e.*, from TCSF) in which different reference genomes (Table 1) were used. Furthermore, we compared the performance of IMRA to that of PRICE, a tool based on a similar idea to IMRA. In these testing procedures, we used two NGS datasets of two endosymbiont genomes (*Blattabacterium cuenoti* strains BPAA and BPAY) that were obtained by sequencing crude DNA isolated from the fat bodies of the strains’ respective host cockroaches. The purity of the target genomes (*i.e.*, the degree to which the library contained a mixture of target and non-target genomes) differed by an order of magnitude: the BPAA and BPAY libraries were 28.5% and 2.4% pure, respectively. Moreover, distinct numbers of reads were input in each of the procedures. Thus, the effects of purity and the depth of coverage of the input data were also examined concomitantly.

### Selection of *de novo* assemblers for the present strategy

In order to determine which assembler was the most appropriate for the present datasets, we made a comparison of the results of *de novo* assembly using the three assembler programs, each implementing distinct algorithms (Table S2). In terms of NGA50 (a statistic representing the quality of the assembly), IDBA-UD showed the highest values in several cases among the three tools tested (0.1–0.25 M reads for the BPAA library; 1 and 5 M reads for the BPAY library). However, we observed several mis-assemblies generated by IDBA-UD when a small number of reads were input (0.1–0.15 and 0.3 M reads for the BPAA library; 1–1.5 M reads for the BPAY library). Regarding the subsequent IMRA step (*i.e.* elongation and merging contigs from selected contigs), accuracy in *de novo* assembly was considered the most important factor for contigs as an initial point of our strategy, and, thus, the use of IDBA-UD for the present datasets was considered inappropriate. In terms of the coverage of a genome (‘Genome fraction’ in Table S2), Newbler gave a better performance than ABySS when a small dataset was used (0.1 and 1 M reads for BPAA and BPAY libraries, respectively). Efficiency in *de novo* assembly from a small dataset is important in a situation with a low-throughput sequencing platform or sequencing of multiplexed libraries (*i.e.*, a small amount of data per library). Overall, we determined Newbler, the OLC type assembler, to be the most suitable assembler for the present dataset, and, thus, decided to incorporate it into the present strategy. Genome assembly computations in the subsequent parts of this study were carried out using Newbler.

### Accuracy and sensitivity of the tested methods of contig selection

**Fig. 2:**
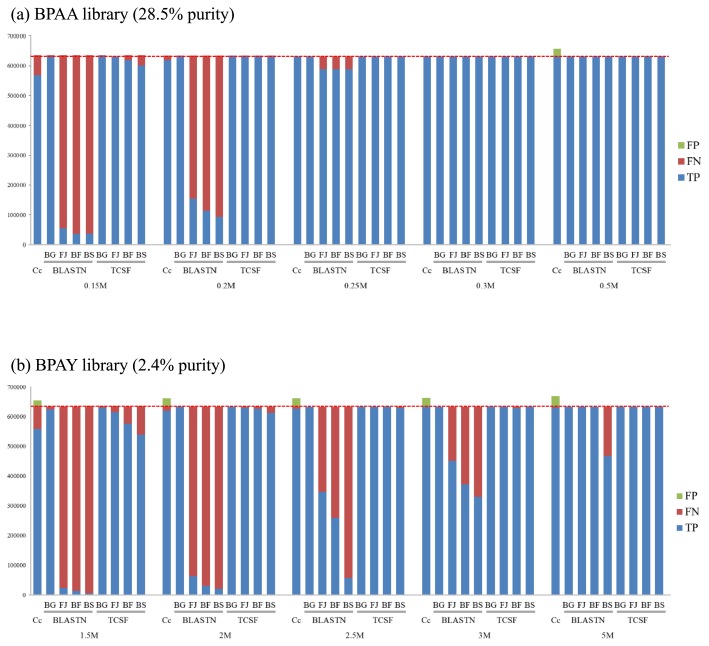
Sensitivity and accuracy in contig selection for *de novo* sequence assembly performed with the use of distinct numbers of input reads for the target endosymbiont genomes of *Blattabacterium cuenoti* strains (a) BPAA and (b) BPAY. Each column denotes the total length of the contigs in a selection that was accurately included (blue part, true positives [TPs]), incorrectly excluded (red part, false negatives [FNs]), and incorrectly included (green part, false positives [FPs]). Abbreviations for the reference genomes: BG, *B. cuenoti* strain BGIGA; FJ, *Flavobacterium johnsoniae*; BF, *Bacteroides fragilis*; BS, *Bacillus subtilis*. Cc, Concoct (a contig-selection method based on the sequence composition and depth of coverage; ref. 15). The broken line indicates the length of the genomic sequence of *B. cuenoti* (approximately 632 kbp). The purities of the libraries (*i.e.*, the fractions of endosymbiont-derived reads in the datasets) were 28.5% and 2.4% for *B. cuenoti* strains BPAA and BPAY, respectively.

The evaluated accuracies and sensitivities of the tested methods of contig selection (Concoct, BLASTN, and TCSF, with distinct reference genomes) are summarized in Fig. 2.

In terms of accuracy, FPs were higher in Concoct, the reference-independent method, than in homology search-based methods (BLASTN and TCSF) in all situations (Fig. 2), except for a few cases in which no FP was recorded by any method (see also Table S3 and S4). In the BPAY library, which was the target genome of low purity, the FPs reached 20,868–35,653 nt (equivalent to 3.3%–5.6% of the full length *B. cuenoti* genome, Fig. 2b; for the values obtained, see Table S3b). In the case of the BPAA library, the target genome of higher purity, markedly lower FPs were obtained in Concoct than in the BPAY library FPs (0%–3.5% of the full length *B. cuenoti* genome, Fig. 2a vs Fig. 2b; see also Table S3a and S4a). In the Concoct cases, the number and total length of FPs were elevated by an increase in the number of input reads used for assembly (Table S3 and S4). On the other hand, only low FPs were observed in the homology search-based methods, BLASTN and TCSF, in all situations tested in this study (<2,485 nt, <0.4% of the sequence length of the *B. cuenoti* genome; Table S3). In the BPAA library, no FP was recorded in either BLASTN or TCSF. Nevertheless, a few FPs were recorded in TCSF in the case of the library with low purity (<2,485 nt of FPs in the BPAY library), and these FPs gradually became higher in terms of total length with increases in the number of input reads (Fig. 2, Table S3); however, they were smaller than those observed in Concoct by an order of magnitude. Performing further BLAST searches against the NCBI NT database showed that the sequences of the FPs observed in TCSF exhibited >99% partial similarity to the bacterial 16S rRNA genes derived from the genus *Serratia* (data not shown), and this was attributed to incidental contamination during experimental procedures such as the dissection of insect tissues.

The sensitivity of the contig selection by homology search-based methods (BLASTN and TCSF) was affected by the number of input reads for the preceding *de novo* assembly as well as the genetic distance between the reference genome and target endosymbiont (Fig. 2). When the most closely related strain (BG in Fig. 2), which belonged to the same species with the target genome, was employed as the reference genome, both of these methods showed very high sensitivity (*i.e.*, low FNs) regardless of the number of input reads. However, when the genomic sequences of free-living relatives (FJ, BF, and BS in Fig. 2) were used as the reference, sensitivity in both methods decreased with increases in the genetic distance from the target genome. Nevertheless, this effect was markedly larger in BLASTN than in TCSF; the difference between the two methods was prominent when a small number of reads was input. In the cases of the BPAA and BPAY libraries, which featured small numbers of input reads (0.15–0.2 and 1.5–3 M reads, respectively), extremely low sensitivity (*i.e.*, high FNs) was observed using BLASTN. In these cases, 76%–95% and 29–99% of the sequence length of the target genome were not covered in the *B. cuenoti* strains BPAA and BPAY, respectively (Fig. 2). However, the sensitivity of this method improved when the number of input reads was increased, and this was attributed to an increase in the input data vastly improving the result of the *de novo* assembly, and thereby diminishing the problem of FNs (see Table S3). In contrast to BLASTN, the adverse effect of the genetic distance on sensitivity was only minor in TCSF. When a closely related free-living bacterium (*Flavobacterium johnsoniae*, FG in Fig. 2) was used as the reference, >97% of the sequence length of the target genome was covered in TCSF regardless of the number of input reads, in spite of the marked difference observed between the reference and target genomes (10-fold difference in genome size and 15% difference in the 16S rRNA gene sequence, see Table 1, Table S1). Moreover, even when the reference genome belonged to a different phylum than the target endosymbiont (*i.e.*, *Bacillus subtilis*, 26% difference in the 16S rRNA gene sequence, BS in Fig. 2), TCSF still showed high sensitivity for the selection with the smallest number of input reads in each library (covering >85% of the target genome, 0.15 and 1.5 M reads in BPAA and BPAY libraries, respectively). Concoct also showed high sensitivity, which was similar to that of TCSF with distantly related reference genomes (Cc vs BF or BS in TCSF, Fig. 2): Concoct exhibited >89% coverage of the target genome in the case of the smallest dataset in either library (66,859 nt of FNs in 0.15 M reads with the BPAA library; 76,180 nt of FNs in 1.5 M reads with the BPAY library, Table S3), and >97% coverage with the larger dataset (Table S3). Overall, the sensitivity of TCSF was the highest among the three methods tested when a related genome from a different class in the same phylum (*i.e.*, *Bacteroides fragilis*, 21% difference in the 16S rRNA gene sequence, BF in Fig. 2) or a more closely related genome was used as the reference.

### Improvement in the result of *de novo* assembly by using IMRA

The performance of IMRA was examined in distinct test cases: IMRA was started with contigs selected (using TCSF) from two libraries of high and low purities (*i.e.*, the BPAA and BPAY libraries) by using four reference genomes with varied genetic distances from the target endosymbiont *B. cuenoti* strains (Table 1).

**Fig. 3:**
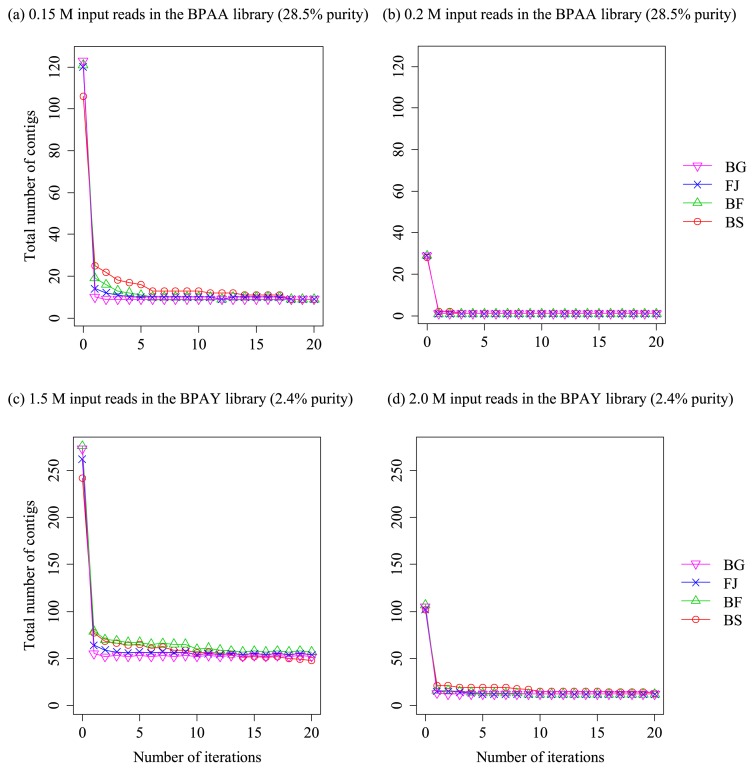
Number of contigs with increasing iterations in the IMRA procedure used with distinct combinations of target and reference genomes. The IMRA procedure was started with filtered contigs obtained using the TCSF procedure with the reference genome. Target endosymbiont genomes and the number of input reads are as follows: (a) *B. cuenoti* strain BPAA (28.5% purity in the DNA library) with 0.15 M input reads; (b) *B. cuenoti* strain BPAA (28.5% purity) with 0.2 M input reads; (c) *B. cuenoti* strain BPAY (2.4% purity) with 1.5 M input reads; and (d) *B. cuenoti* strain BPAY (2.4% purity) with 2 M input reads. Abbreviations for reference genomes used in the preceding TCSF procedure: BG, *B. cuenoti* strain BGIGA; FJ, *Flavobacterium johnsoniae*; BF, *Bacteroides fragilis*; BS, *Bacillus subtilis*.

**Fig. 4:**
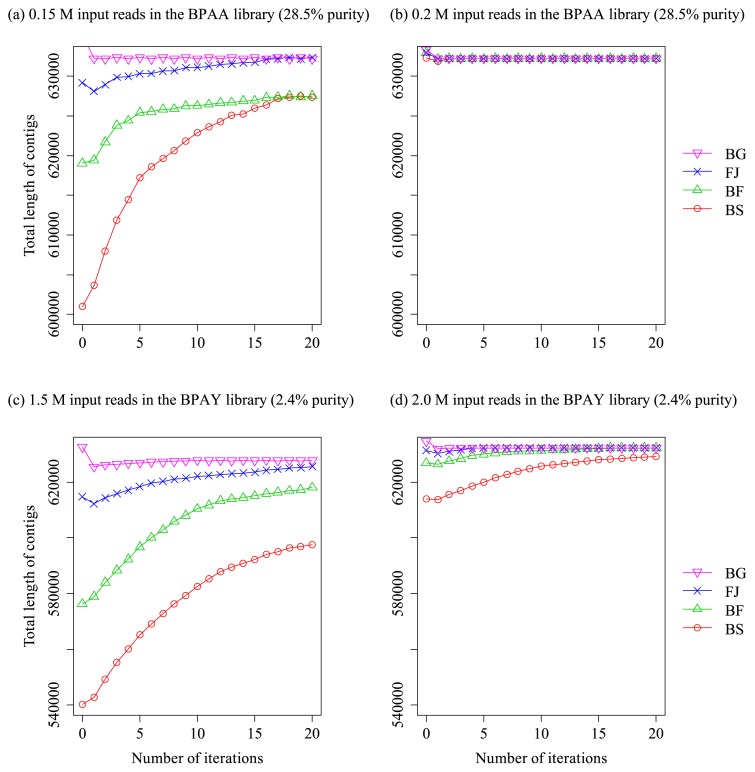
Total length of contigs with increasing iterations in the IMRA procedure used with distinct combinations of target and reference genomes. The IMRA procedure was started with filtered contigs obtained following the use of the TCSF procedure with the reference genome. Target endosymbiont genomes and the number of input reads are as follows: (a) *B. cuenoti* strain BPAA (28.5% purity in the DNA library) with 0.15 M input reads; (b) *B. cuenoti* strain BPAA (28.5% purity) with 0.2 M input reads; (c) *B. cuenoti* strain BPAY (2.4% purity) with 1.5 M input reads; and (d) *B. cuenoti* strain BPAY (2.4% purity) with 2 M input reads. Abbreviations for reference genomes used in the preceding TCSF procedure: BG, *B. cuenoti* strain BGIGA; FJ, *Flavobacterium johnsoniae*; BF, *Bacteroides fragilis*; BS, *Bacillus subtilis*.

With increasing iterations, the statistics of the assemblies (*i.e.*, number and total length of contigs) improved in the BPAA and BPAY libraries (Fig. 3 and 4). In all tested cases of libraries, input data sizes, and reference genomes, the following common pattern of variations in the statistics with increasing iterations was observed: After the first iteration, the number of contigs markedly decreased (Fig. 3) and the total length of contigs decreased concurrently (Fig. 4; in some cases with distantly related reference genomes, the total length was not decreased, but its increase was less than those observed in the subsequent iterations). The total length of contigs then gradually increased toward the full length of the genomic sequence, whereas the number of contigs decreased with increasing iterations.

When large numbers of reads were input (≥0.2 and ≥2.5 M reads for the BPAA and BPAY libraries, respectively), the contigs merged to 3 or less and the total length of the contigs finally reached the full length of the target genomes (Fig. 3b, d and Fig. 4b, d), indicating that the entire genomic region was successfully constructed. When a small dataset was used in both libraries (0.15 and ≤1.5 M reads for the BPAA and BPAY libraries, respectively), the elongations of contigs were saturated before reaching the full length of the genomes; nevertheless, the total length of the contigs increased with iterations until saturation was reached in all cases (Fig. 4; for detailed statistics, see Table S6).

Differences between the reference genomes used to select contigs by means of TCSF, which was used before the IMRA iterations, only exerted an effect when small numbers of reads were input (0.15 and ≤2 M reads for BPAA and BPAY, respectively): In situations in which a free-living relative (FJ, BF, or BS in Fig. 4) was used as the reference genome, IMRA was started with a smaller coverage of the target genome by the contigs than in cases in which a close relative was used as the reference genome (*B. cuenoti* strain BGIGA, BG in Fig. 4). This was because of the small, but notable difference in the sensitivity of TCSF between the four reference genomes when a small number of reads was input (BG, FJ, BF, and BS in TCSF with 0.15 and 1.5 M reads for the BPAA and BPAY libraries shown in Fig. 2, respectively). However, this difference diminished with increases in the iterations of IMRA: Assembly results obtained after IMRA were identical when *F. johnsoniae* (15% difference in the 16S rRNA gene sequence from the target genome) or a more closely related genome was used as the reference. However, this procedure resulted in inferior results when the two other more distant reference genomes were used; however, IMRA markedly improved the assembly results (BF and BS in Fig. 3a, c and Fig. 4a, c).

In order to examine the significance of IMRA, we compared the performance of IMRA with that of PRICE, a similar method previously proposed (36) (Table S5). The results obtained showed that, after the same number of iterations, a larger total contig length and smaller number of contigs were obtained using IMRA than PRICE in all cases tested, with different input data sizes and software settings. Furthermore, elongation of the total length of contigs and decreases in the number of contigs reached saturation in fewer iterations using IMRA than when using PRICE. These results indicated that IMRA improved the genomic assemblies more efficiently than PRICE. In cases with parameter set A (*i.e.* when Newbler’s default values were applied for two parameters common to both methods), markedly larger reductions were observed in the total contig length (~ 244 k nt) during the first iterations in PRICE than during those in IMRA (~ 2.5 k nt), with the likely cause being PRICE requiring more iterations than IMRA before saturation. The drop in total contig length during the first iteration in PRICE was smaller in cases using parameter set B (*i.e.*, the default values of PRICE were applied) than in those using parameter set A, while the performance of IMRA was affected less by parameter sets. Overall, IMRA showed better performance than PRICE regardless of input data sizes and examined parameter sets.

### Performance of the strategy described herein in assembling the genomic sequences of *B. cuenoti* strains BPAA and BPAY

As discussed in the preceding subsections, when more than a certain number of reads were input, the use of TCSF and IMRA improved the assembly of genomic sequences to the point where only a few of the contigs present had a total length equivalent to the genome sizes. Contigs derived from the BPAA library were merged into a single contig in the four cases featuring ≥0.2 M reads. In the case of the BPAY library, contigs obtained from ≥2.5 M reads were finally merged into three or fewer contigs. Furthermore, after trials using different settings, we found a parameter set with which IMRA successfully merged the contigs from TCSF into a single contig from 3M reads (minimum overlap length: ml=45; Table S6).

The genome of *B. cuenoti* strain BPAY, which was newly sequenced in this study, was completely assembled based on the contigs after proofreading the repetitive regions by performing Sanger sequencing. The sequence was validated by manually examining it for the possible presence of chimeric and misaligned reads (data not shown). Furthermore, similarities in the sequence and in the gene set in closely related genomes were determined to be plausible (data not shown).

When we used an NGS dataset obtained using a target genome of only 2.4% purity (*i.e.*, the BPAY library), a single contig of 632,334 bp, which was almost as long as the complete genomic sequence (632,370 bp), was successfully reconstructed by means of the strategy presented herein without the use of any closely related reference genomes. Based on these results, the minimal number of reads required to complete the genomic sequence of a *B. cuenoti* strain using the strategy presented here was estimated to be approximately 18× depth of the coverage for the target genome, regardless of the purity of the dataset.

## Discussion

In the present study, we developed a procedure to select contigs of a target genome from an output of *de novo* assembly based on similarities in the translated sequences to a reference genome (TCSF); this procedure exhibited higher sensitivity and accuracy than a reference-independent method (Concoct) when a free-living bacterium belonging to the same phylum (or closer) with the target endosymbiont was used as the reference. The performance of TCSF was relatively stable regardless of the reference genome (*i.e.*, upon using the genome of either a closely related strain or a free-living distant relative); in contrast, the sensitivity of a nucleotide sequence-based method (BLASTN) decreased when a free-living relative (belonging to a different or more distant family) was used as a reference. Furthermore, iterative filtering of a dataset by mapping the reads on the selected contigs to reassembled contigs obtained from the filtered reads (IMRA) greatly improved sequence assembly by means of elongating and merging the contigs. Thus, by using a strategy consisting of TCSF and IMRA, the genomic sequences of two target endosymbionts (*B. cuenoti* strains BPAA and BPAY) were almost completely assembled from the datasets obtained by sequencing crude DNA samples, regardless of the purity of the samples. Moreover, compared with previously reported methods, in the strategy described herein, a markedly smaller depth of coverage was required for completing a *Blattabacterium* genome, which indicated the extremely high efficiency of this strategy (as discussed later in this section).

### Comparison of methods used for sequence selection for endosymbionts

The statistical information in contigs (*e.g.*, sequence composition and/or depth of coverage) is considered to serve as clues that help distinguish the sequences of an endosymbiont from other sequences (18). The G+C content (one way to evaluate sequence composition) has been utilized as a clue (18, 39) because the genomes of endosymbionts are typically highly biased toward A+T (*e.g.*, *B. cuenoti* strains feature 24%–28% G+C content; ref. 13, 23, 29, 32, 37, 39, 46), whereas the depth of coverage has also been utilized as the clue (18, 37, 39) mainly because a host cell often harbors large numbers of endosymbiont cells, which results in the copy number of the endosymbiont genomes being markedly higher than that of the host genome (22, 45). Therefore, these characteristics appeared to be appropriate for use as the benchmark in separating the contigs of endosymbionts from an admixture of host and symbiont contigs. However, with respect to the base composition, overlaps between those of the contigs derived from endosymbionts and other genomes were allowed because host genomes vary widely in base compositions depending on the position on the chromosomes, and also because mitochondrial genomes are typically biased toward A+T base compositions, as are the genomes of endosymbionts in invertebrates. Furthermore, when a DNA library of low purity is sequenced for the target endosymbiont, the dataset that contains, in the large part, sequences derived from genomes other than the target endosymbiont, may result in an increase in the occurrence of overlaps in the distribution of sequence composition as well as the depth of coverage between them. These reasons may explain why Concoct, a method based on statistical information for the sequence composition (although it utilizes the k-mer composition instead of the G+C content) and depth of coverage, selected a higher number of invalid contigs than the other methods when Concoct was applied to the low-purity library (the BPAY library). In our strategy, it is important to minimize false positives in contig selection from *de novo* assembly because the selected contigs are to be elongated in the subsequent step (IMRA). From this viewpoint, Concoct was not preferable to the other methods examined in the present study, although it showed sufficiently high sensitivity.

The discrimination of contigs based on sequence similarities with relevant organisms may be more accurate and less susceptible to experimental variables, such as the concentration of an endosymbiont’s DNA in a library, than the contig selection with Concoct. We demonstrated that a nucleotide-based BLAST method (BLASTN) used with the genome sequence of a closely related endosymbiont strain exhibited high sensitivity and accuracy regardless of the purity of the library. Nevertheless, as also shown by our results, the performance of BLASTN in terms of sensitivity depended highly on the genetic distance between the target and reference genomes; however, this problem was minor when a DNA sample of high purity was used (*i.e.*, in the case of the BPAA library). These results indicate that using the BLASTN method is not practical when sequencing an endosymbiotic bacterium whose close relative’s genomes have not yet been sequenced.

In contrast to the aforementioned methods, TCSF, the translated sequence-based method developed in this study, demonstrated high sensitivity and accuracy in the contig selection regardless of library purity. In addition, its performance was affected less by the reference genome used, whereas the use of more closely related reference genomes resulted in better performance. TCSF exhibited the highest sensitivity among the methods tested, even when the genome of a free-living relative, which belongs to a different class in the same phylum with the target genome, was used as the reference genome. This result supports the view that the genomes of obligate endosymbionts, which are often highly reduced in size, are strictly inherited as a part of the genome of their common ancestor (27). Furthermore, most genes involved in central cellular processes (*e.g.*, replication, transcription, and translation, ref. 27) are generally conserved in symbiotic and free-living bacteria, which explains the wide range of applicabilities of variable reference genomes for this method. In terms of accuracy, certain contigs derived from the mitochondrial genome and 18S rRNA genes of the host genome were incorrectly included immediately after selecting the sequences by using TBLASTX (data not shown). Therefore, we equipped an additional step in TCSF to filter out such contigs by using available sequences derived from related host species. Consequently, high sensitivity and accuracy were both achieved using TCSF, as shown in the results presented herein.

There are several tools that have the ability to align contigs generated from *de novo* assembly onto a reference genome (*e.g.*, ABACAS [2], Projector2 [48], and OSLay [35]). The development of these tools has been motivated by the demand to design PCR primer pairs for gap closing for unfinished genomes. These tools may be used in the contig selection of an endosymbiont genome in place of our TCSF. Nevertheless, given that any closely related reference genomes are not available and/or the result of a *de novo* assembly is poor (*i.e.* its contigs are very short), their sensitivity in retrieving valid contigs may be poorer than that with BLAST-based methods such as TCSF. This is because these methods were designed to detect overall synteny between a contig and reference sequence rather than to find local homology between sequences; however, a user can optionally use BLAT (17) as a search algorithm in some tools (*e.g.* ABACAS, which uses MUMmer by default). In addition to this difference, TCSF was implemented with features specialized for endosymbiont genomes, *i.e.* procedures to eliminate contigs derived from mitochondrial and host genomic rRNA genes).

### Effects of the iterative procedure of reference mapping and re-assembling on sequence assembly

**Fig. 5:**
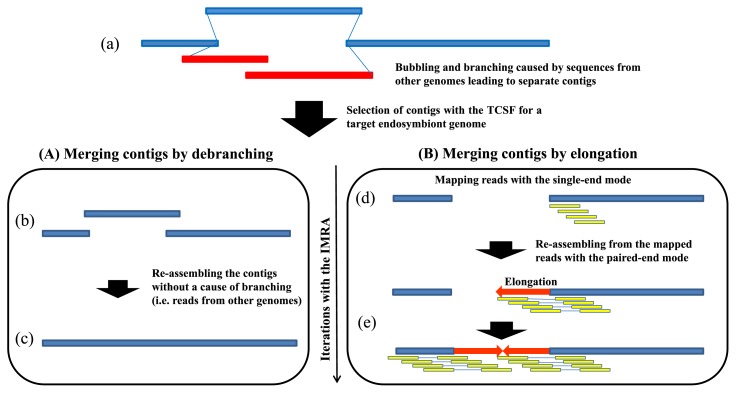
A schematic view of the improved genomic-sequence assembly following the use of TCSF and IMRA procedures in the strategy developed in this study. (a) A genomic sequence separated into contigs as a result of the path branching caused by sequences derived from other genomes. (b) A set of contigs obtained after removing contigs derived from other genomes. (c) Sequences merged into a contig by reassembling only reads that aligned onto the contigs. (d) Mapping reads onto contigs featuring a gap between them by using the single-end mode. (e) Elongating the contig ends by reassembling the mapped reads to make their unmapped-ends join the contig; the iterations of such an elongation close the gap.

The use of IMRA successfully improved sequence assembly by means of the iterative reassembling performed using filtered reads, and this led to the elongation of contigs and gap closing. This procedure appeared to allow us to use a library more effectively (Table S6). As indicated by the scheme presented in Fig. 5, IMRA helped improve sequence assembly in two ways: through the merging of contigs by removing invalid reads causing the branching of a path in mathematical graphs constructed using the assembling software (debranching, Fig. 5c), and by closing gaps by means of elongating contig edges (elongation, Fig. 5e).

In this study, we observed a marked reduction in the total number of contigs at the first iteration of IMRA, which indicated that these contigs merged into larger contigs. Since the total length of the contigs was also decreased concurrently, the observed improvement may have occurred as a result of the effects of debranching rather than to the elongation of contigs. A large fraction of the invalid reads obtained from other genomes may have been filtered out by means of mapping onto the selected contigs, and reassembling performed without these reads may have output a sequence assembly that was markedly better than the previous assembly.

The effect of contig elongation appeared when IMRA was started with a set of contigs that insufficiently covered the target genome. (*i.e.*, with the use of 0.15 M reads of input in Fig. 4a, 1.5–2 M reads in Fig. 4c and d). In these cases, a gradual increase in the total length of contigs toward the full length of the target genomes and a gradual decrease in the number of contigs were observed with increasing iterations. This effect almost compensated for the difference in genome coverage by the contigs, which arose because of distinct reference genomes being used during TCSF (Fig. 4a, c, and d). In other words, the performance of IMRA was only slightly affected by lower genome coverage beforehand, which was caused by the reduced selectivity (albeit a slight reduction) of TCSF when a distantly related genome was used as a reference sequence. When the input numbers of reads were insufficient (0.15 and 1.5–2 M reads for BPAA and BPAY libraries, respectively), the procedure presented herein could not be used to complete the target genome sequences because the reads corresponding to the remaining gaps were absent in the dataset. Nevertheless, even in such cases, the merging of contigs through debranching and elongation obtained using IMRA may greatly reduce the labor and time costs required for gap closing (Table S6).

Several programs utilizing paired-end reads, such as GapFiller (5), IMAGE (47), and PRICE (36), have been developed to improve the genomic assembly output of *de novo* assembly tools. While the former two close gaps in scaffolds, PRICE improves the genomic assembly, similar to our IMRA, by the elongation and merging of the selected contigs. PRICE was developed to assemble viral genomes and transcripts from metagenomic datasets, while IMRA was developed for endosymbiont genomes. PRICE finds paired-reads, one of the members of which may be (fully or partially) aligned onto the terminus of a contig, and then performs assembly from the reads to extend the contig. After extension, it merges two (or more) of the contigs when they are fully overlapped. PRICE iteratively performs these steps, while IMRA improves an assembly in every iteration by reassembling entire contigs from read-pairs, at least one member of which is (fully or partially) mapped onto the previous contigs.

Comparisons of the results obtained from IMRA and PRICE on the same datasets showed that IMRA was superior to PRICE in terms of performance in all tested cases (Table S5). This may have been due to the distinct algorithms used in the software: De Bruijn graph- and OLC-based algorithms in PRICE and Newbler (which is used in IMRA), respectively. The latter algorithm generally works better for sequence data with a low depth of coverage on the target genome (25), as shown in the comparison between *de novo* assemblers in the present study (*i.e.*, ABySS vs Newbler in Table S2). This may have resulted in the difference observed in performance between PRICE and IMRA. Moreover, when IMRA cannot complete a genome, Newbler outputs scaffolds, in which some of the contigs are ordinated. These characteristics may be helpful for gap closing.

In terms of the accuracy of IMRA, we found that it suppressed the retrieval of contigs from non-target genomes in most cases. Although contigs from other genomes were incorrectly (albeit not abundantly) collected by TCSF and were input to IMRA (Table S3 and S4), it did not extend such sequences. This may have been because IMRA filters out small contigs (shorter than 500 nt using the default settings) after assembly in every iteration. That is, the FP contigs from TCSF, most of which were small, were assembled in the first iteration in IMRA and most of them remained shorter than 500 nt. After this, IMRA discarded them. An exception was when a large number of reads were input from a low-purity library (*i.e.*, 5 M reads from the BPAY library). In this case, an FP contig from the 16S rRNA gene of another bacterium (assumed to be a strain belonging to the genus *Serratia*) was not eliminated by IMRA because its initial length was nearly 500 nt, and subsequently exceeded this after the first iteration (546 nt). When a stricter cut-off threshold (1,000 nt) was applied, such a contig was not observed in the output (Table S5). These results indicate that filtering small contigs with an appropriate threshold value prevented sequences from other genomes being elongated.

We also observed that, during every iteration in IMRA, reads from other genomes that had high, albeit partial, similarity to the target genome were included (again, assumed to be sequences from the 16S rRNA gene of the bacterium described above). Nevertheless, no contig assembled from such reads was found in the outputs. In the present cases, the mean insert sizes in the DNA libraries were approximately 230 nt, while the threshold value for the cut-off length was more than twice as large. Logically, a contig newly assembled from such invalid reads was unlikely to exceed 500 nt in length after the initial iteration. Therefore, it may be important for the accuracy of IMRA to determine a contig size cut-off threshold value according to the insert size of the DNA library.

A comparison of the sequence-assembling results described here with those obtained in previous studies, in which the genomes of other *B. cuenoti* strains were sequenced, revealed that we successfully obtained the largest contigs among these studies (Table 2), and these contigs were very close to the full length of the genomes. Moreover, compared with other studies in which the same NGS platform (*i.e.*, Illumina) was employed as the one we used, we obtained considerably more favorable results with lower numbers of reads being input. For example, in the case of the genome of *B. cuenoti* strain MADAR (39), reads were sequenced to an 83× depth of coverage and then assembled into 93 contigs, whereas the results here showed that, by using our strategy, a *Blattabacterium* genome was completed from, at a minimum, 18× depth of coverage of reads. These results indicated that the efficiency of our strategy was higher than that of other methods previously used in studies on *Blattabacterium* genomes.

The favorable results achieved in the present study were likely due, at least in part, to the simple structures of *Blattabacterium* genomes, which only harbor a few multicopy genes and repetitive elements (32). In the case of more complex genomes, namely those in free-living bacteria, facultative symbionts, and recently-evolved obligate symbionts, the final genomic assembly may contain separate contigs even after being processed by our strategy, because such genomes contain multiple copies of ribosomal RNA genes, transposon-derived sequences, and tandem repeats. Nevertheless, our strategy may greatly improve genome assembly in such cases by reducing gapped fragments more effectively than previous strategies, and may also contribute to studies on such genomes.

### Prospects of using our strategy in further applications

In this study, we developed a strategy to assemble the sequences of *B. cuenoti* genomes; this strategy features a novel approach that exploits the traits of the genome evolution of this endosymbiont (32), and we demonstrated that the performance of this approach was superior to those of methods described in previous studies. Our results showed that the genomic sequences of a distant relative (belonging to a different class in the same phylum with the target genome or more closely related) may be used as a reference for sequence assembly, suggesting that this strategy is applicable to other bacterial endosymbionts, even if the genome sequence of a closely-related strain has been unavailable. Conversely, the potential limitations of the strategy presented here are as follows: If two or more distinct, but mutually related endosymbionts co-exist in the tissue of a host organism, discriminating between their sequences may prove challenging. Another potential challenge may be encountered if an endosymbiont has acquired several genes from its host or from other co-inhabiting bacteria by means of horizontal transfer events; however, such cases are rare in endosymbionts (9, 14) and have not been reported in the primary endosymbionts of invertebrates (9, 27). Nevertheless, by presuming that the traits of conserved genome evolution revealed in *B. cuenoti* strains are universal among endosymbiotic bacteria (26, 27, 32, 44), our method may be applied to a wide range of endosymbionts. Since the methods used in this strategy were very simple, but the procedure was a little complicated, its automation by our scripts may lead to the use of this strategy in a wide range of research, including areas outside bioinformatics. Moreover, because of the efficiency achieved coupled with a small data size, this method may provide researchers with an opportunity to concurrently analyses multiple genomes of endosymbiotic bacteria, even when using a low-throughput sequencing platform. Therefore, the results presented herein may contribute substantially to the development of this study field.

## Supplementary Information



## Figures and Tables

**Table 1:** Taxonomical and genomic information of bacterial strains employed as target and reference genomes in the present study.

	Target genome		Reference genome			
		
	*Blattabacterium cuenoti* BPAA	*Blattabacterium cuenoti* BPAY	*Blattabacterium cuenoti* BGIGA	*Flavobacterium johnsoniae* UW101	*Bacteroides fragilis* 638R	*Bacillus subtilis natto* BEST195
Gram stain	Negative	Negative	Negative	Negative	Negative	Positive
Phylum	*Bacteroidetes*	*Bacteroidetes*	*Bacteroidetes*	*Bacteroidetes*	*Bacteroidetes*	*Firmicutes*
Class	*Flavobacteriia*	*Flavobacteriia*	*Flavobacteriia*	*Flavobacteriia*	*Bacteroidia*	*Bacilli*
Order	*Flavobacteriales*	*Flavobacteriales*	*Flavobacteriales*	*Flavobacteriales*	*Bacteroidales*	*Bacillales*
Family	*Blattabacteriaceae*	*Blattabacteriaceae*	*Blattabacteriaceae*	*Flavobacteriaceae*	*Bacteroidaceae*	*Bacillaceae*
Lifestyle	Intracellular symbiont	Intracellular symbiont	Intracellular symbiont	Free-living	Free-living	Free-living
Genome size	0.6 M	0.6 M	0.6 M	6.3 M	5.4 M	4.1 M
Number of CDS	575	573	572	5017	4290	4375
Number of rRNA operons	1	1	1	6	6	10
GC content (%)	26.4	26.3	25.7	34.1	43.4	43.6
Accession number	NC_020510	AP_014609	NC_017924	NC_009441	NC_016776	NC_017196
Abbreviation*	BPAA	BPAY	BG	FJ	BF	BS
Identity to target**	—	—	98%	83–84%	79%	74%

* Abbreviations used for strains in the present study.

** Sequence identity in 16S ribosomal RNA genes between target and reference bacteria (see Table S1 for more detailed information).

**Table 2:** Statistics of sequencing and assembly computations of *Blattabacterium cuenoti* strains in this study and previous studies.

Strain	BPLAN	BGE	MADAR	CPU	BGIGA	BOR	BPAA	BPAY
Host organisms	*Periplaneta americana*	*Blattella germanica*	*Mastotermes darwiniensis*	*Cryptocercus punctulatus*	*Blaberus giganteus*	*Blatta orientalis*	*Panesthia angustipennis spadica*	*Panesthia angustipennis yayeyamensis*
Genome size (Mb)	0.63	0.63	0.59	0.6	0.63	0.63	0.63	0.63
Literature	Sabree *et al.*(36)	Lopez *et al.*(22)	Sabree *et al.*(38)	Neef *et al.*(28)	Huang *et al.*(12)	Patino *et al.*(31)	This study	This study
Sequencing platform	Roche 454 GS FLX	a 454 platform (model unknown)	Illumina Genome Analyzer IIx	a 454 platform (model unknown)	Illumina Genome Analyzer IIx	Roche 454 GS FLX	Illumina HiSeq 2000	Illumina HiSeq 2000
Isolation of endosymbiont cells	Yes	yes	no	Yes	no	Yes	no	no
Number of input reads (PE†)	0	0	19,264,920	0	17,458,144	0	200,000*	3,000,000*
Number of input reads (SE†)	225,047	NA	59,294	709,186	205,533	133,562	0	0
Mean length of reads (nt)	225	NA	100	224 and 344	100	235	100	100
Purity of DNA library (%)‡	49	NA	2.7	28.3	NA	97	28.5	2.4
Number of contigs	13	NA	93	43	40	39	1**	1**

† PE and SE indicate paired-end reads and single-end reads, respectively.

‡ Estimated as the proportion of input reads aligned onto contigs in percentages.

* A part of the obtained dataset was input; the two libraries were multiplexed and sequenced using single-lane runs on a sequencer.

** Assembly computations were performed according to the procedure described in Fig. 1 by using the genomic sequence of *Bacteroides fragilis* (NC_016776, ref. 32) as the reference genome.

## References

[1] Asahina S (1988). Taxonomic notes on Japanese Blattaria: XVII. The species of the genus *Panesthia*. Med Entomol Zool.

[2] Assefa S, Keane TM, Otto TD, Newbold C, Berriman M (2009). ABACAS: Algorithm-based automatic contiguation of assembled sequences. Bioinformatics.

[3] Bandi C, Sironi M, Damiani G, Magrassi L, Nalepa CA, Laudani U, Sacchi L (1995). The establishment of intracellular symbiosis in an ancestor of cockroaches and termites. Proc R Soc Lond B.

[4] Blochmann F (1887). Vorkommen bakterienaehnliche Koerpechen in den Geweben and Eiern verschiederen Insecten. Biol Zentralbl.

[5] Boetzer M, Pirovano W (2012). Toward almost closed genomes with GapFiller. Genome Biol.

[6] Brooks MA (1970). Comments on classification of intracellular symbiotes of cockroaches and description of the species. J Invertebr Pathol.

[7] Buchner P (1965). Endosymbiosis of Animals with Plant Microorganisms.

[8] Camacho C, Coulouris G, Avagyan V, Ma N, Papadopoulos J, Bealer K, Madden TL (2009). BLAST+: Architecture and applications. BMC Bioinform.

[9] Dunning Hotopp JC (2011). Horizontal gene transfer between bacteria and animals. Trends Genet.

[10] Gier HT (1936). The morphology and behavior of the intracellular bacteroids of roaches. Biol Bull.

[11] Gil R, Silva FJ, Zientz E (2003). The genome sequence of *Blochmannia floridanus*: comparative analysis of reduced genomes. Proc Natl Acad Sci USA.

[12] Gurevich A, Saveliev V, Vyahhi N, Tesler G (2013). QUAST: quality assessment tool for genome assemblies. Bioinformatics.

[13] Huang CY, Sabree ZL, Moran NA (2012). Genome sequence of *Blattabacterium* sp. strain BGIGA, endosymbiont of the *Blaberus giganteus* cockroach. J Bacteriol.

[14] Husnik F, Nikoh N, Koga R (2013). Horizontal gene transfer from diverse bacteria to an insect genome enables a tripartite nested mealybug symbiosis. Cell.

[15] Iorizzo M, Senalik D, Szklarczyk M, Grzebelus D, Spooner D, Simon P (2012). *De novo* assembly of the carrot mitochondrial genome using next generation sequencing of whole genomic DNA provides first evidence of DNA transfer into an angiosperm plastid genome. BMC Plant Biol.

[16] Johannes A, Bjarnason BS, de Bruijn I, Schirmer M, Quick J, Ijaz UZ, Lahti L, Loman NJ, Andersson AF, Quince C (2014). Binning metagenomic contigs by coverage and composition. Nat Methods.

[17] Kent WJ (2002). BLAT—the BLAST-like alignment tool. Genome Res.

[18] Kumar S, Jones M, Koutsovoulos G, Clarke M, Blaxter M (2013). Blobology: exploring raw genome data for contaminants, symbionts and parasites using taxon-annotated GC-coverage plots. Front Genet.

[19] Kurtz S, Phillippy A, Delcher AL, Smoot M, Shumway M, Antonescu C, Salzberg SL (2004). Versatile and open software for comparing large genomes. Genome Biol.

[20] Lanham UN (1968). The Blochmann bodies: Hereditary intracellular symbionts of insects. Biol Rev Camb Philos Soc.

[21] Lo N, Bandi C, Watanabe H, Nalepa C, Beninati T (2003). Evidence for cocladogenesis between diverse Dictyopteran lineages and their intracellular endosymbionts. Mol Biol Evol.

[22] Lopez-Sanchez MJ, Neef A, Patino-Navarrete R, Navarro L, Jimenez R, Latorre A, Moya A (2008). Blattabacteria, the endosymbionts of cockroaches, have small genome sizes and high genome copy numbers. Environ Microbiol.

[23] Lopez-Sanchez MJ, Neef A, Pereto J, Patino-Navarrete R, Pignatelli M, Latorre A, Moya A (2009). Evolutionary convergence and nitrogen metabolism in *Blattabacterium* strain Bge, primary endosymbiont of the cockroach *Blattella germanica*. PLoS Genet.

[24] McBride MJ, Xie G, Martens EC (2009). Novel features of the polysaccharide-digesting gliding bacterium *Flavobacterium johnsoniae* as revealed by genome sequence analysis. Appl Environ Microbiol.

[25] Miller JR, Koren S, Sutton G (2010). Assembly algorithms for next-generation sequencing data. Genomics.

[26] Moran NA (1996). Accelerated evolution and Muller’s rachet in endosymbiotic bacteria. Proc Natl Acad Sci USA.

[27] Moran NA, Bennett GM (2014). The tiniest tiny genomes. Ann Rev Microbiol.

[28] Nakabachi A, Yamashita A, Toh H, Ishikawa H, Dunbar HE, Moran NA, Hattori M (2006). The 160-kilobase genome of the bacterial endosymbiont *Carsonella*. Science.

[29] Neef A, Latorre A, Peretó J, Silva FJ, Pignatelli M, Moya A (2011). Genome economization in the endosymbiont of the wood roach *Cryptocercus punctulatus* due to drastic loss of amino acid synthesis capabilities. Genome Biol Evol.

[30] Nishito Y, Osana Y, Hachiya T, Popendorf K, Toyoda A, Fujiyama A, Itaya M, Sakakibara Y (2010). Whole genome assembly of a natto production strain *Bacillus subtilis natto* from very short read data. BMC Genomics.

[31] Paszkiewicz K, Studholme D (2010). *De novo* assembly of short sequence reads. Brief Bioinform.

[32] Patino-Navarette R, Moya A, Latorre A, Pereto J (2013). Comparative genomics of *Blattabacterium cuenoti*: the frozen legacy of an ancient endosymbiont genome. Genome Biol Evol.

[33] Patrick S, Blakely GW, Houston S (2010). Twenty-eight divergent polysaccharide loci specifying within- and amongst-strain capsule diversity in three strains of *Bacteroides fragilis*. Microbiol.

[34] Peng Y, Leung HCM, Yiu SM, Chin FYL (2012). IDBA-UD: a de novo assembler for single-cell and metagenomic sequencing data with highly uneven depth. Bioinformatics.

[35] Richter DC, Schuster SC, Huson DH (2007). OSLay: optimal syntenic layout of unfinished assemblies. Bioinformatics.

[36] Ruby JG, Bellare P, DeRisi JL (2013). PRICE: Software for the targeted assembly of components of (Meta) Genomic sequence data. G3 (Bethesda).

[37] Sabree ZL, Kambhampati S, Moran NA (2009). Nitrogen recycling and nutritional provisioning by *Blattabacterium*, the cockroach endosymbiont. Proc Natl Acad Sci USA.

[38] Sabree ZL, Degnan PH, Moran NA (2010). Chromosome stability and gene loss in cockroach endosymbionts. Appl Environ Microbiol.

[39] Sabree ZL, Huang CY, Arakawa G, Tokuda G, Lo N, Watanabe H, Moran NA (2012). Genome shrinkage and loss of nutrient-providing potential in the obligate symbiont of the primitive termite *Mastotermes darwiniensis*. Appl Environ Microbiol.

[40] Sanger F, Nicklen S, Coulson AR (1977). DNA sequencing with chain-terminating inhibitors. Proc Natl Acad Sci USA.

[41] Schatz MC, Delcher AL, Salzberg SL (2010). Assembly of large genomes using second-generation sequencing. Genome Res.

[42] Shigenobu S, Watanabe H, Hattori M, Sakaki Y, Ishikawa H (2000). Genome sequence of the endocellular bacterial symbiont of aphids *Buchnera* sp. APS. Nature.

[43] Simpson JT, Wong K, Jackman SD, Schein JE, Jones SJ, Birol I (2009). ABySS: A parallel assembler for short read sequence data. Genome Res.

[44] Tamas I, Klasson L, Canback B, Naslund AK, Eriksson AS, Wernegreen JJ, Sandstrom JP, Moran NA, Andersson SG (2002). 50 million years of genomic stasis in endosymbiotic bacteria. Science.

[45] Tokuda G, Lo N, Takase A, Yamada A, Hayashi Y, Watanabe H (2008). Purification and partial genome characterization of the bacterial endosymbiont *Blattabacterium cuenoti* from the fat bodies of cockroaches. BMC Res Notes.

[46] Tokuda G, Elbourne LDH, Kinjo Y (2013). Maintenance of essential amino acid synthesis pathways in the *Blattabacterium cuenoti* symbiont of a wood-feeding cockroach. Biol Lett.

[47] Tsai IJ, Otto TD, Berriman M (2010). Improving draft assemblies by iterative mapping and assembly of short reads to eliminate gaps. Genome Biol.

[48] van Hijum SAFT, Zomer AL, Kuipers OP, Kok J (2005). Projector 2: contig mapping for efficient gap-closure of prokaryotic genome sequence assemblies. Nucleic Acids Res.

