# Supplementary material for: An Efficient Strategy Developed for Next-Generation Sequencing of Endosymbiont Genomes Performed Using Crude DNA Isolated from Host Tissues: A Case Study of Blattabacterium cuenoti Inhabiting the Fat Bodies of Cockroaches
Source: Microbes Environ. 2015 Jul 4;30(3):208–20. doi: 10.1264/jsme2.ME14153 (PMC4567559; doi:10.1264/jsme2.ME14153)
Supplement: Supplementary file 1 [file 30_208_s1.pdf]

Supplemental file 1

Softwares and parameters used for quality assessment
of our strategy in present study

----

1. Software utilized

FastX Tool kit ver. 0.0.13
URL: http://hannonlab.cshl.edu/fastx_toolkit/

cmpfastq_pe
URL: http://compbio.brc.iop.kcl.ac.uk/software/cmpfastq_pe.php

Newbler ver. 2.9
URL: http://www.454.com/products/analysis-software/

ABySS ver. 1.3.4
Reference: Simpson, J.T., et al. (2009)
URL: http://www.bcgsc.ca/platform/bioinfo/software/abyss

IDBA-UD ver. 1.1.1
Reference: Peng, Y., et al. (2012)
URL: http://i.cs.hku.hk/~alse/hkubrg/projects/idba_ud/

2. Settings for software

Parameters for software specified in present study are listed below.
Only additional parameters are listed and specifications for input
and/or output files are omitted.

2.1 Quality control for the raw reads.

<FastX>
```
fastq_quality_filter  -q 27 -p 80 -Q 33
fastq_quality_trimmer -t 27 -l 80 -Q 33
```

<Cmpfastq_pe>
```
perl cmpfastq_pe.perl
```

2.2 Performance comparison between three de novo assembler software

<Newbler>
```
runAssembly   -large
```

<ABySS>
```
abyss-pe       k=60
```

<IDBA-UD>
```
fq2fa         --merge  read_1.fq read_2.fq read.fa
idba_ud       -r read.fa
```

2.3 Performance comparison of contig selection.

<TCSF>
```
TCSF.bash     -e1 1e-12 -e2 1e-12
```

<BLASTN>
```
blastn        -evalue 1e-12
              -outfmt 6
              -num_alignment 1
```

2.4 Performance comparison between IMRA and PRICE with 2 parameter sets.

<IMRA>

(parameter set A)
```
IMRA.bash     -n 20
```

(parameter set B)
```
IMRA.bash     -n 20
              -miA 85
              -miM 85
```

```
               -mlA 35
               -mlM 35

<PRICE>
(parameter set A)
 PriceTI       -fp read_1.fq read_2.fq 250
               -icf contigs.fa 1 1 1
               -mol 40 -mpi 90
               -nc 20

(parameter set B)
 PriceTI       -fp read_1.fq read_2.fq 250
               -icf contigs.fa 1 1 1
               -nc 20
```

(a) Sequence identities between the genomes

	BPAA	BPAY	BNCIN	BGIGA	BGE	BOR	BPLAN	CPU
BPAY	98.62							
BNCIN	87.46	87.42						
BGIGA	88.01	88.01	87.29					
BGE	84.75	84.71	84.45	84.80				
BOR	83.38	83.25	83.33	83.21	83.33			
BPLAN	83.44	83.36	83.35	83.34	83.55	97.07		
CPU	83.11	83.17	83.20	83.26	83.42	84.13	84.26	
MADAR	83.15	83.09	83.20	83.26	83.41	84.17	84.03	85.05

(b) Sequence identities between 16S rRNA genes

	BPAA	BPAY	BNCIN	BGIGA	BGE	BPLAN	BOR	CPU	MADAR	FJ*	BF*
BPAY	99.67										
BNCIN	98.03	97.96									
BGIGA	97.90	97.96	97.44								
BGE	97.38	97.83	97.11	97.18							
BOR	96.26	96.20	96.26	95.67	95.67						
BPLAN	95.70	95.91	95.57	95.29	95.64	98.40					
CPU	95.28	95.38	95.40	95.54	95.93	95.41	94.80				
MADAR	94.58	94.51	94.24	94.30	94.86	94.86	94.37	95.28			
FJ*	83.14	83.60	83.14	82.81	83.01	82.55	81.73	82.68	81.26		
BF*	79.17	79.32	79.08	79.27	79.17	78.38	77.71	78.73	77.16	80.31	
BS*	74.06	73.53	73.96	74.42	74.06	73.47	72.87	73.01	73.35	75.24	72.14

Table S1. Sequence identities of (a) the whole genomes and (b) 16S rRNA genes between *Blattabacterium cuenoti* strains and free-living relatives. Values shown are percentages. Sequence identity for a pair of strains was calculated based on alignment by using the NUCmer module in the MUMmer software package (11) and by using ClustalW alignments (http://www.clustal.org/clustal2/) for whole genomes and 16S rRNA genes, respectively. See Tables 1 and 2 for scientific names of free-living species (FJ, BF, and BS; indicated by asterisks) and the host organism of *B. cuenoti* strains (BPAA - MADAR), respectively.

(a) *Blattabacterium cuenoti* strain BPAA (632,490bp; 28.5% of purity in the DNA library)

Input reads	Total length*			Number of contigs*			NGA50			Genome fraction (%)			Number of misassemblies		
	Abyss	IDBA-UD	Newbler	Abyss	IDBA-UD	Newbler	Abyss	IDBA-UD	Newbler	Abyss	IDBA-UD	Newbler	Abyss	IDBA-UD	Newbler
0.1M	642648	583196	636645	1183	401	636	828	1739	1280	97.330	92.052	98.783	0	8	0
0.15M	635193	626722	635618	346	119	139	3055	8433	5311	99.373	99.012	99.906	0	2	0
0.2M	633463	632170	633329	72	29	31	17092	42669	19569	99.926	99.907	99.967	0	0	0
0.25M	632853	632285	632221	14	8	4	101273	333949	101769	99.997	99.952	99.949	0	0	0
0.3M	632567	632649	632305	2	5	1	518794	333949	632305	100.000	99.982	99.989	0	1	0
0.5M	632463	632391	632192	1	1	2	632463	632391	485600	99.995	99.984	99.952	0	0	0

(b) *Blattabacterium cuenoti* strain BPAY (632,370bp; 2.4% of purity in the DNA library)

Input reads	Total length*			Number of contigs*			NGA50			Genome fraction (%)			Number of misassemblies		
	Abyss	IDBA-UD	Newbler	Abyss	IDBA-UD	Newbler	Abyss	IDBA-UD	Newbler	Abyss	IDBA-UD	Newbler	Abyss	IDBA-UD	Newbler
1M	591105	481924	594770	1676	552	973	428	793	776	88.891	78.203	92.171	0	8	0
1.5M	642693	596877	633899	781	287	353	1272	2577	2974	98.419	95.143	99.113	0	2	0
2M	640033	623983	634195	315	124	117	3483	7065	10390	99.665	98.539	99.820	0	0	0
2.5M	635971	631333	633070	103	44	31	12550	24845	35434	99.959	99.743	99.858	0	0	0
3M	633755	632297	632447	40	15	12	29608	86325	97337	99.975	99.942	99.860	0	0	0
5M	632521	632447	632287	2	1	3	322519	632447	165585	99.983	99.994	99.978	0	0	0

Table S2. Performance of de novo assembly by the assembler tools tested in the present study. Assembly statistics were calculated by using QUAST (11) with the completed genomic sequence as the reference for each library. * The total length and number of contigs that were aligned onto the respective reference sequences. For the definitions of NGA50 and Genome fraction, see text.

(a) *Blattabacterium cuenoti* strain BPAA (632,490bp; 28.5% of purity in the DNA library)

Number of input reads	Total length of contigs (FP/FN)								
	Concoct	BLASTN				TCSF			
		BG	FJ	BF	BS	BG	FJ	BF	BS
0.15M	570,459 (1,700/66,859)	630,964 (0/4,654)	55,538 (0/580,080)	37,414 (0/598,204)	36,699 (0/598,919)	635,191 (0/427)	629,199 (0/6,419)	619,048 (0/16,570)	601,042 (0/34,576)
0.2M	618,919 (0/14,410)	633,176 (0/153)	155,014 (0/478,315)	115,486 (0/517,843)	93,081 (0/540,248)	633,329 (0/0)	632,984 (0/345)	632,984 (0/345)	632,281 (0/1,048)
0.25M	632,221 (0/0)	632,221 (0/0)	587,384 (0/44,837)	587,384 (0/44,837)	587,384 (0/44,837)	632,221 (0/0)	632,221 (0/0)	632,221 (0/0)	632,221 (0/0)
0.3M	632,305 (0/0)	632,305 (0/0)	632,305 (0/0)	632,305 (0/0)	632,305 (0/0)	632,305 (0/0)	632,305 (0/0)	632,305 (0/0)	632,305 (0/0)
0.5M	655,471 (23,279/0)	632,192 (0/0)	632,192 (0/0)	632,192 (0/0)	632,192 (0/0)	632,192 (0/0)	632,192 (0/0)	632,192 (0/0)	632,192 (0/0)

(b) *Blattabacterium cuenoti* strain BPAY (632,370bp; 2.4% of purity in the DNA library)

Number of input reads	Total length of contigs (FP/FN)								
	Concoct	BLASTN				TCSF			
		BG	FJ	BF	BS	BG	FJ	BF	BS
1.5M	579,080 (20,868/76,180)	624,316 (0/10,076)	23,214 (0/611,178)	13,504 (0/620,888)	5,069 (159/629,482)	632,550 (364/2,206)	614,702 (159/19,849)	576,346 (0/58,046)	540,349 (364/94,497)
2M	646,572 (26,298/13,921)	633,738 (0/457)	62,569 (0/571,626)	29,608 (0/604,587)	20,610 (159/613,744)	634,640 (445/0)	631,284 (159/3,070)	626,871 (0/7,324)	613,910 (445/20,730)
2.5M	655,689 (28,571/6,054)	633,172 (0/0)	347,782 (0/285,390)	258,496 (0/374,676)	57,027 (159/576,304)	633,840 (668/0)	633,409 (382/145)	633,250 (223/145)	629,174 (668/4,666)
3M	663,254 (30,807/0)	632,447 (0/0)	450,835 (316/181,928)	371,456 (0/260,991)	330,541 (475/302,381)	633,431 (984/0)	633,145 (698/0)	629,514 (708/3,641)	633,431 (984/0)
5M	667,940 (35,653/461)	632,748 (0/0)	633,565 (817/0)	632,748 (0/0)	467,534 (371/165,585)	634,013 (1,265/0)	634,021 (1,273/0)	634,642 (1,894/0)	635,233 (2,485/0)

Table S3. Sensitivity and accuracy of the examined methods used for selecting contigs for the genomes of the target endosymbiont *Blattabacterium cuenoti* strains (a) BPAA and (b) BPAY. BG, FJ, BF and BS indicate reference genomes used in the selections (see Table 1 for details). Values are total length of contigs (nt). FP: false positives, incorrectly included contigs that were derived from other genomes by the selection process. FN: false negatives, incorrectly excluded contigs that were derived from the target genome by the selection process. The FP and FN values are presented in parentheses. The total length of the genome of *B. cuenoti* strain BPAA or BPAY is approximately 632 kbp. The purities of the libraries (*i.e.,* the fractions of endosymbiont-derived reads in the datasets) were 28.5% and 2.4% for *B. cuenoti* strains BPAA and BPAY, respectively.

(a) *Blattabacterium cuenoti* strain BPAA (632,490bp; 28.5% of purity in the DNA library)

Number of input reads	Number of contigs (FP/FN)								
	Concoct	BLASTN				TCSF			
		BG	FJ	BF	BS	BG	FJ	BF	BS
0.15M	83 (1/57)	128 (0/11)	9 (0/130)	7 (0/132)	5 (0/134)	138 (0/1)	129 (0/10)	121 (0/18)	106 (33/0)
0.2M	23 (0/8)	30 (0/1)	9 (0/22)	7 (0/24)	5 (0/26)	31 (0/0)	29 (0/2)	29 (0/2)	28 (0/3)
0.25M	4 (0/0)	4 (0/0)	3 (0/1)	3 (0/1)	3 (0/1)	4 (0/0)	4 (0/0)	4 (0/0)	4 (0/0)
0.3M	1 (0/0)	1 (0/0)	1 (0/0)	1 (0/0)	1 (0/0)	1 (0/0)	1 (0/0)	1 (0/0)	1 (0/0)
0.5M	9 (7/0)	2 (0/0)	2 (0/0)	2 (0/0)	2 (0/0)	2 (0/0)	2 (0/0)	2 (0/0)	2 (0/0)

(b) *Blattabacterium cuenoti* strain BPAY (632,370bp; 2.4% of purity in the DNA library)

Number of input reads	Number of contigs (FP/FN)								
	Concoct	BLASTN				TCSF			
		BG	FJ	BF	BS	BG	FJ	BF	BS
1.5M	209 (9/154)	322 (0/34)	15 (0/341)	10 (0/346)	7 (1/350)	355 (2/3)	315 (1/42)	276 (0/80)	242 (2/116)
2M	105 (12/24)	114 (0/3)	15 (0/102)	12 (0/105)	7 (1/111)	119 (2/0)	111 (1/7)	107 (0/10)	102 (2/17)
2.5M	39 (13/6)	32 (0/0)	10 (0/22)	8 (0/24)	7 (1/26)	35 (3/0)	33 (2/1)	32 (1/1)	32 (3/3)
3M	38 (16/0)	12 (0/0)	6 (1/7)	4 (0/8)	5 (2/9)	16 (4/0)	15 (3/0)	14 (3/1)	16 (4/0)
5M	21 (18/1)	4 (0/0)	6 (2/0)	4 (0/0)	4 (1/1)	8 (4/0)	8 (4/0)	10 (6/0)	13 (9/0)

Table S4. Sensitivity and accuracy of the examined methods used for selecting contigs for the genomes of the target endosymbiont *Blattabacterium cuenoti* strains (a) BPAA and (b) BPAY. BG, FJ BF and BS indicate reference genomes used in the selections (see Table 1 for details). Values are number of contigs. FP: false positives, incorrectly included contigs that were derived from other genomes by the selection process. FN: false negatives, incorrectly excluded contigs that were derived from the target genome by the selection process. The FP and FN values are presented in parentheses. The total length of the genome of *B. cuenoti* strain BPAA or BPAY is approximately 632 kbp. The purities of the libraries (*i.e.*, the fractions of endosymbiont-derived reads in the datasets) were 28.5% and 2.4% for *B. cuenoti* strains BPAA and BPAY, respectively.

(a) *Blattabacterium cuenoti* strain BPAA (632,490bp; 28.5% of purity in the DNA library)

Iteration	0.15M								0.2M							
	Number of contigs				Total length				Number of contigs				Total length			
	Parameter set A		Parameter set B		Parameter set A		Parameter set B		Parameter set A		Parameter set B		Parameter set A		Parameter set B	
	PRICE	IMRA	PRICE	IMRA	PRICE	IMRA	PRICE	IMRA	PRICE	IMRA	PRICE	IMRA	PRICE	IMRA	PRICE	IMRA
0	120(129)	120(129)	120(129)	120(129)	629199	629199	629199	629199	29	29	29	29	632984	632984	632984	632984
1	50(63)	14	42(52)	14	397869	628111	568804	628720	13(14)	1	7	2	600374	632222	621119	632246
2	51(102)	12	41(69)	11	407153	628967	571303	629511	12(19)	1*	7(9)	2*	601588	632243*	621616	632249*
3	52(110)	11	40(69)	9	414352	629861	574971	630324	12(22)		7(10)		602925		622138	
4	60(111)	10	45(67)	9	421313	629972	578094	630861	15(20)		8(9)		604068		622641	
5	72(109)	10	49(64)	9	427880	630328	580840	631157	17(20)		9		605178		623093	
6	76(105)	10	51(59)	9	434016	630362	583267	631493	18(20)		9		606388		623564	
7	79(106)	10	51(59)	9	440495	630667	585750	631866	18(20)		9		607453		623984	
8	81(103)	10	51(60)	9	446167	630716	588329	632190	18(21)		9		608648		624474	
9	80(104)	10	51(62)	8	452167	631091	591000	632211	17(21)		8		609255		624383	
10	80(97)	10	51(58)	8	457916	631116	593012	632318	17(22)		8		610198		624547	
11	78(96)	10	50(57)	8*	463888	631269	595077	632232*	18(20)		8		610905		624667	
12	78(92)	9	50(58)		469135	631485	597205		17(19)		8		611769		624879	
13	78(99)	10	50(60)		474584	631532	598950		16(18)		8		612143		625042	
14	76(96)	10	50(61)		479283	631718	600717		16(18)		8		612592		625206	
15	76(95)	10	50(61)		483822	631769	602755		16(18)		8		613137		625425	
16	74(90)	10	48(55)		487119	632136	603644		16(18)		8		613617		625559	
17	73(89)	10	47(54)		490881	632205	604505		16(18)		8		614159		625767	
18	72(90)	9*	47(55)		494713	632351*	605553		16(18)		8		614684		626004	
19	72(89)		47(54)		498350		606540		16(19)		8		615150		626170	
20	72(90)		47(54)		501844		607508		16(19)		8		615596		626339	

Table S5. Improving performance of genomic assembly with iterations of IMRA and PRICE for libraries (a) BPAA and (b) BPAY with two parameter sets. Parameter set A, 90 and 40 for minimum overlap identity and minimum overlap length, respectively (the default values in Newbler); parameter set B, 85 and 35 (the default values in PRICE); * iteration after which improvement of an assembly reached saturation. 'Number of contigs' represents those ≥500 nt and, in parentheses, those ≥100 nt. ** For the case of 2M in the BPAY library, Newbler did not work with parameter set B; the settings were changed slightly to avoid this problem (using a minimum overlap length of ml = 36 instead of 35).

(b) *Blattabacterium cuenoti* strain BPAY (632,370bp; 2.4% of purity in the DNA library)

Iteration	1.5M								2M							
	Number of contigs				Total length				Number of contigs				Total length			
	Parameter set A		Parameter set B		Parameter set A		Parameter set B		Parameter set A		Parameter set B**		Parameter set A		Parameter set B**	
	PRICE	IMRA	PRICE	IMRA	PRICE	IMRA	PRICE	IMRA	PRICE	IMRA	PRICE	IMRA	PRICE	IMRA	PRICE	IMRA
0	262(315)	262(315)	262(315)	262(315)	614702	614702	614702	614072	102(113)	102(111)	102(113)	102(111)	631284	631284	631284	631284
1	141(202)	64	135(181)	67	468137	612262	533887	613760	50(62)	15	43(52)	16	462780	630279	610069	630424
2	137(277)	59	118(197)	60	483168	614313	538797	616378	48(84)	15	39(58)	16	467671	630943	607562	631922
3	138(300)	57	118(203)	61	493314	615834	543986	617870	49(88)	14	38(57)	15	470740	631503	608518	631843
4	141(291)	56	121(195)	59	501195	617061	547777	618815	51(84)	13	39(55)	14	473753	632107	609273	632272
5	155(294)	57	128(199)	62	508109	618343	551747	620845	59(85)	12	41(54)	14	477315	632188	610267	632268
6	167(295)	56	133(196)	58	514541	619592	555182	621291	62(87)	12*	43(55)	15*	480015	632191*	611380	632886*
7	173(290)	57	137(193)	62	519981	620214	558405	622917	64(84)		43(54)		482695		612384	
8	176(287)	56	138(193)	58	525317	621002	561822	623434	64(82)		43(53)		485188		613213	
9	175(282)	57	138(193)	60	529799	621424	564654	623852	65(87)		43(52)		488261		614013	
10	175(279)	54	137(195)	57	534043	622070	567127	626039	65(85)		43(52)		490983		614700	
11	175(275)	55	138(197)	60	537411	622278	569356	625687	65(80)		43(52)		493445		615337	
12	175(281)	54	137(198)	56	540481	622707	571222	626938	65(81)		43(52)		495914		616017	
13	176(281)	55	137(194)	57	543117	623045	572832	626148	65(85)		43(52)		498576		616639	
14	176(281)	54	137(195)	55	545396	623225	574436	627419	64(79)		43(52)		500682		617230	
15	175(280)	55	137(193)	57	547633	623602	575818	626423	64(81)		43(52)		502811		617680	
16	176(282)	54	139(195)	55	549730	624257	577357	627627	62(80)		43(52)		504278		618090	
17	175(281)	55	138(195)	57	551451	624551	578542	626610	61(80)		43(52)		505371		618570	
18	174(276)	54	138(195)	55*	552643	625017	579712	627775*	61(79)		43(51)		506496		618926	
19	174(274)	55	137(197)		553842	625149	580892		62(79)		43(51)		507579		619309	
20	174(274)	54	136(194)		555128	625515	581837		61(78)		43(51)		508505		619619	

Table S5 (continued).

(a) *Blattabacterium cuenoti* strain BPAA (632,490bp; 28.5% of purity in the DNA library)

Number of input reads	Total length of contigs (bp)			Number of contigs		
	De novo assembly	After TCSF	After IMRA	De novo assembly	After TCSF	After IMRA
0.15M	876,711	619,048	627,531	172(1,219)	114(121)	9
0.2M	957,120	632,984	632,243	98(1,429)	29(29)	1
0.25M	1,033,318	632,221	632,390	98(1,723)	4(4)	1
0.3M	1,112,940	632,305	632,305	126(2,040)	1(2)	1
0.5M	1,443,534	632,284	632,284	231(3,457)	2(2)	1

(b) *Blattabacterium cuenoti* strain BPAY (632,370bp; 2.4% of purity in the DNA library)

Number of input reads	Total length of contigs (bp)			Number of contigs		
	De novo assembly	After TCSF	After IMRA	De novo assembly	After TCSF	After IMRA
1.5M	3,822,049	576,346	619,344	1,103(14,186)	233(276)	54
2M	4,816,916	626,871	632,191	1,117(18,572)	99(107)	12
2.5M	5,770,256	633,250	632,368	1,295(22,965)	30(32)	3
3M	6,749,382	629,514	632,282	1,424(27,553)	11(14)	2[1]*
5M	10,911,092	634,642	634,327 [632,225]*	2,148(47,899)	3(10)	2[1]*

Table S6. Improvement of the *de novo* sequence assembly of *Blattabacterium cuenoti* strains (a) BPAA and (b) BPAY achieved by using the presented strategy comprising TCSF and IMRA. Numbers of contigs are of those ≥500 nt and of those including ≥100 nt (in parentheses). *De novo* assembly was performed using Newbler (Ver. 2.9; paired-end mode); TCSF was performed by using the genomic sequence of *Bacteroides fragilis* (NC_016776, Ref. 32) as the reference. The purities of the libraries (*i.e.,* the fractions of endosymbiont-derived reads in the datasets) were 28.5% and 2.4% for *B. cuenoti* strains BPAA and BPAY, respectively. * Values in square brackets were those from runs with modified parameter sets (minimum overlap length, ml = 45 for 3M; cut-off contig size, l = 1000 for 5M).
